# Extraskeletal Myxoid Chondrosarcoma: State of the Art and Current Research on Biology and Clinical Management

**DOI:** 10.3390/cancers12092703

**Published:** 2020-09-21

**Authors:** Silvia Stacchiotti, Giacomo Giulio Baldi, Carlo Morosi, Alessandro Gronchi, Roberta Maestro

**Affiliations:** 1 Medical Oncology Unit 2, Cancer Medicine Department, Fondazione IRCCS Istituto Nazionale Tumori, 20133 Milan, Italy; 2 “Sandro Pitigliani” Medical Oncology Department, Hospital of Prato, 59100 Prato, Italy; giacomogiulio.baldi@uslcentro.toscana.it; 3 Deparment of Radiology, Fondazione IRCCS Istituto Nazionale Tumori, 20133 Milan, Italy; carlo.morosi@istitutotumori.mi.it; 4 Department of Surgery, Fondazione IRCCS Istituto Nazionale Tumori, 20133 Milan, Italy; alessandro.gronchi@istitutotumori.mi.it; 5 Unit of Oncogenetics and Functional Oncogenomics, Centro di Riferimento Oncologico di Aviano (CRO) IRCCS, National Cancer Institute, 33081 Aviano, Italy; rmaestro@cro.it

**Keywords:** sarcoma, chondrosarcoma, NR4A3 fusions, chemotherapy, antiangiogenics

## Abstract

**Simple Summary:**

The aim of this review is to provide an overview of the biological basis of pathogenesis and current research in extraskeletal myxoid chondrosarcoma (EMC), together with the state of the art of treatment for localized and advanced disease. EMC is an ultra-rare sarcoma sub-type, more often arising from the soft tissues, marked by specific molecular features consisting in rearrangement of the *NR4A3* gene, identified in recent years and very useful to distinguish EMC from other mimics. Available pharmacological treatments in particular are discussed, with a focus on the most recent results and future perspectives.

**Abstract:**

Extraskeletal myxoid chondrosarcoma (EMC) is an ultra-rare mesenchymal neoplasm with uncertain differentiation, which arises mostly in the deep soft tissue of proximal extremities and limb girdles. EMC is marked by a translocation involving the *NR4A3* gene, which can be fused in-frame with different partners, most often *EWSR1* or *TAF1*. Although EMC biology is still poorly defined, recent studies have started shedding light on the specific contribution of NR4A3 chimeric proteins to EMC pathogenesis and clinical outcome. Standard treatment for localized disease is surgery, plus or minus radiation therapy with an expected prolonged survival even though the risk of relapse is about 50%. In advanced cases, besides the standard chemotherapy currently used for soft tissue sarcoma, antiangiogenic agents have recently shown promising activity. The aim of this review is to provide the state of the art of treatment for localized and advanced disease, with a focus on pharmacological treatments available for EMC. The biological basis of current research and future perspectives will be also discussed.

## 1. Introduction

Extraskeletal myxoid chondrosarcoma (EMC) is an ultra-rare sarcoma subtype with an incidence of <1/1,000,000 inhabitants/year. It usually occurs in adults, with a median age of onset in the fifth decade and only a few cases have been reported in childhood and adolescence [1,2]. The male/female ratio is 2:1. Most EMCs arise in the deep soft tissue of the proximal extremities and limb girdles, with the thigh being the most common primary site [3,4]. EMC may also occur in less common sites such as the trunk, head and neck, paraspinal soft tissue, abdomen, retroperitoneal space [5,6], and bone [7,8,9]. Originally thought to be a cartilaginous neoplasm, EMC is now classified as a mesenchymal tumor of uncertain differentiation [1]. Indeed, EMC exhibits distinctive clinico-pathological and genetic features while it shows no cartilage differentiation, despite the name, which has been retained only for historical reasons [1,10]. This review aims at providing an overview on current knowledge on EMC biology and the state-of-the-art treatment for localized and advanced disease, with a focus on pharmacological treatments available for EMC and an eye on future perspectives.

## 2. Diagnostic Criteria

Symptoms at disease onset are related to the site of origin. Often, EMC is characterized by an enlarged, deep-seated soft tissue mass, accompanied by pain and tenderness; some tumors may mimic a hematoma and, when located in close proximity to joints, they can cause functional impairment. EMC usually appears as a lesion with a low attenuated density in computed tomography (CT) scans and hyper-intense signaling with hypo-intense internal septa lesion in T2-weighted magnetic resonance (MR) imaging; pronounced lobular architecture is often seen. Hemorrhagic and necrotic degeneration can also be found and signal characteristics in T1-weighted MR vary accordingly, from low- to intermediate- and high-intensity signals [11,12,13]. Metastases are usually pulmonary, however, extra-pulmonary metastases can occur, with lymph node involvements seen more often than in other soft tissue sarcoma (STS) subtypes [14,15].

### Pathological and Molecular Characteristics

**Figure 1:**
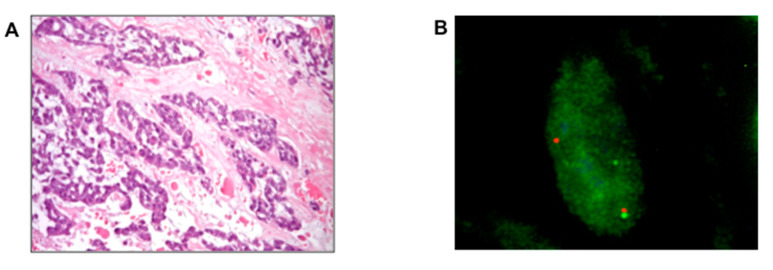

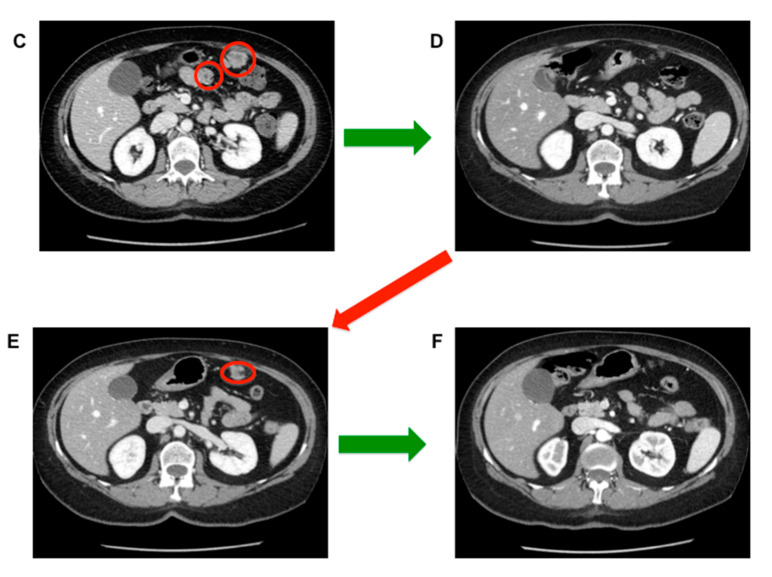
Response to sunitinib in a patient affected by intraperitoneal metastases from an extraskeletal myxoid chondrosarcoma (EMC) primary of the thigh. (**A**) Hematoxylin and eosin stain of extraskeletal myxoid chondrosarcoma arising from the thigh; (**B**) Fluorescence in situ hybridization FISH analysis confirming *NR4A3* rearrangement (split signals: centromere, orange; telomere, green, in contrast to normal fused signals); (**C**) Peritoneal metastases (red circles) treated with sunitinib 37.5 mg/day with complete response (green arrow; no more tumor lesion detectable in (**D**) maintained over 2 years. After two years of treatment, sunitinib was discontinued with evidence of progression (red arrow) after 8 months from discontinuation, as confirmed by the appearance of a new peritoneal lesion ((**E**), red circle). A new complete response was achieved after rechallenging sunitinib (green arrow, no tumor lesion in (**F**)).

Histopathologically, EMC is characterized by an abundant hypo-cellular myxoid matrix and interconnecting cords of uniform neoplastic cells with a common spindle cell differentiation. Neoplastic cells can interconnect to form small clusters and complex trabecular or cribriform arrays. Well-formed hyaline cartilage is virtually never seen and the mitotic rate is usually low (Figure 1A,B).

High-grade, dedifferentiated variants of EMC are described [16,17] with areas of increased cellularity, decreased myxoid matrix, and undifferentiated solid areas with epithelioid cytomorphology. The expression of synaptophysin and neuron-specific enolase (NSE) is present in some cases, suggesting a possible neural/neuroendocrine differentiation [18,19,20,21].

From a genetic standpoint, EMC is typified by a reciprocal chromosome translocation involving the *Nuclear Receptor Subfamily 4 Group A (NR4A3)* gene on chromosome 9q31.1 (previously mapped to 9q22), in a near-diploid karyotype. Stenman and coworkers reported this translocation in 1995 for the first time [22]. Gene fusions involving *NR4A3* and leading to *NR4A3* constitutive expression have to date been described exclusively in EMC and are therefore considered a hallmark of this disease. Accordingly, the last version of the World Health Organization (WHO) classification of soft tissue and bone tumors released in 2020 has introduced the alternative provisional definition of “NR4A3-rearranged myxoid sarcoma” for EMC [1]. The positivity for *NR4A3* rearrangements may be particularly helpful in the differential diagnosis of EMC mimics such as myoepithelioma and myoepithelial carcinoma [23].

**Figure 2:**
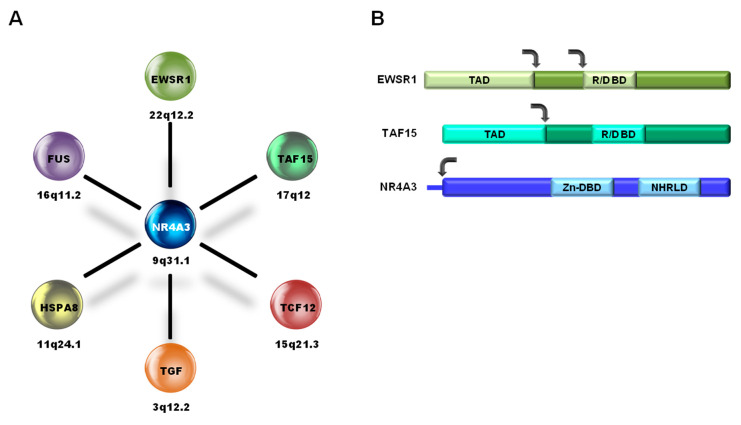
(**A**) *NR4A3* fusion partners in EMC and corresponding chromosome locations; (**B**) schematic representation of the protein regions involved in the most common EMC fusions, *EWSR1-NR4A3* (>70% of all fusions) and *TAF15-NR4A3* (~20% of all fusions). TAD, transactivation domain; R/D BD, RNA/DNA-binding domain; Zn-DBD, zinc finger DNA-binding domain; NHRLD, nuclear hormone receptor-like domain. The arrows indicate the most common breakpoint regions.

Typically, in EMC translocations, *NR4A3* breaks in proximity to the ATG start site and translocates, with its whole coding sequence, downstream of the partner gene that, in general, contributes with a strong promoter and part of the N-terminus (Figure 2). In the vast majority of cases (over 70%), *NR4A3* fuses to the N-terminal transactivation domain of *Ewing Sarcoma RNA Binding Protein 1* (*EWSR1*) (chromosome 22q12.2), giving rise to the EWSR1-NR4A3 chimeric protein; less frequently (about 20% of cases) to the transactivation domain of *TATA-Box Binding Protein Associated Factor 15 (TAF15)* (aliases *Npl3, RBP56, TAF2N, TAFII68*), located on 17q12. Rarer (<5%) *NR4A3* fusion partners include *FUS* (alias *TLS*), *TCF12* (aliases *ALF1, HEB, HTF4, ME1, REB, bHLHb20*), and *Transforming Growth Factor* (*TFG*) (aliases *HMSNP, SPG57, TF6, TRKT3*) [24,25,26,27]. A case of EMC carrying a novel pathogenetic *Heat Shock Protein Family A Member 8 (HSPA8)-NR4A3* fusion was recently reported in which *NR4A3* is placed under the control of the strong promoter of the heat shock protein HSPA8 [28].

The major fusion partners of NR4A3 in EMC, namely EWSR1 and TAF15, are members of the FUS/TLS, EWSR1, and TAF15 (FET/TET) family of RNA/DNA-binding proteins that participate in the control of transcription and RNA/miRNA processing [29]. FET/TET proteins are highly expressed factors that contribute to the generation of a number of oncogenic gene fusions also implicated in other sarcoma subtypes [30]. The FET/TET N-terminus not only confers transactivation activity but is implicated in a number of macromolecular interactions, thus providing the fusion protein with unique biochemical properties [29].

NR4A3 (aliases CHN, CSMF, MINOR, NOR1, TEC) belongs to the NR4A family of nuclear hormone (steroid/thyroid) receptors that also includes NR4A1/NURR77 and NR4A2/NURR1. Despite having a C-terminal nuclear hormone receptor-like domain (NHRLD), NR4A proteins are considered orphan receptors as no known endogenous ligand has been identified. Recent reports point at unsaturated fatty acids as possible ligands [31,32]. NR4A proteins have transcriptional regulatory functions. In fact, through the centrally located zinc finger DNA-binding domain (Zn-DBD), they regulate in a tissue-specific manner the transcription of a wide set of genes involved in key biological functions such as proliferation, apoptosis, DNA repair, metabolism, and differentiation [33]. A growing body of evidence suggests a role for NR4A protein dysfunction in cancer, and in certain tumors, cytosolic localization seems to be associated with aggressive behavior [31,34].

As a result of the translocation events, *NR4A3* becomes constitutionally expressed in EMC. However, the specific biological properties of the diverse *NR4A3* fusion variants are poorly defined. Recently, it was reported that *EWSR1*- and *TAF15*-translocated EMCs feature a different transcriptional profile, with the axon guidance pathway being a major discriminant. In particular, compared to the *EWSR1-NR4A3* EMC subtype, *TAF15-NR4A3* EMC over-expresses pro-tumorigenic axonal guidance molecules, supporting the notion that the type of *NR4A3* partner dictates an axon guidance switch that affects tumor biology [35]. These findings are in agreement with some reports suggesting that *TAF15-NR4A3* EMC features a more aggressive phenotype, with a high-grade morphology and plasmocytoid/rhabdoid morphology in over half of *TAF15*-translocated EMCs [36]. The expression of axon guidance molecules, such as semaphorins and plexins, discloses a potential employment of these molecules as therapeutic targets. In addition, the axon guidance pathway is known to establish crosstalk with REarranged during Transfection (RET) and vascular endothelial growth factor (VEGFR) signaling [37,38], and RET and components of VEGFR pathways are robustly expressed in a significant fraction of EMCs [39,40,41]. These findings are particularly intriguing in the light of the therapeutic activity of antiangiogenic drugs observed in EMC [39,40,42,43,44]. Wingless-related integration site (WNT) and MYC pathways may represent additional therapeutic targets, as key molecules of these signaling pathways have been described to be over-expressed in EMC compared to other sarcomas [45]. The same holds true for peroxisome proliferator-activated receptor gamma (PPARG) and Serum/Glucocorticoid Regulated Kinase 1 (SGK1), which have been demonstrated to be transcriptional targets of the oncogenic fusion protein [10,46].

Besides the pathognomonic translocation, secondary abnormalities seem to occur quite randomly. EMC shows a very low mutation burden with no recurrent gene mutation pattern [41]. Few relatively recurrent secondary chromosomal aberrations have been reported, among which is a copy number gain of 1q25-qter and 8q and trisomy of chromosome 12 [26]. Additional copies of chromosome 7 and 19, as well as losses of chromosome 6q and 8p, have also been reported [41,47]. Panagopoulos and coworkers noticed that the pattern of secondary events in EMC is very similar to that described in Ewing’s sarcoma and suggested that these two mesenchymal tumors may rely on common pathways for malignant progression [26].

Finally, preliminary data on the immune infiltrate of EMC showed lymphoid and myeloid infiltration, mostly localized in the peri-tumoral areas, while intra-tumoral areas were rich in myeloid cells (tumor-associated macrophages) [40].

## 3. State of the Art: Localized Disease

Wide local resection with negative microscopic margins is the standard therapeutic approach for localized disease, as for all patients with an adult type localized STS [48,49]. Local recurrence rate after a wide excision is, however, higher than the usual rate after surgery for STS, ranging from 35% to 50% at 5 years [3,4,14,16,17,50]. Prognostic factors for local recurrences are size, previous unplanned excision, and lack of radiation therapy (RT) administration.

**Figure 3:**
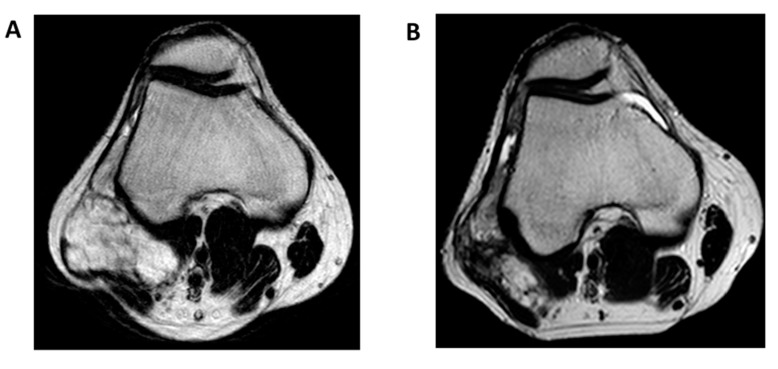
Response to radiation therapy in extraskeletal myxoid chondrosarcoma of the knee. (**A**) T2-weighted magnetic resonance imaging, axial view, at baseline; (**B**) T2-weighted magnetic resonance imaging, axial view, after external beam radiotherapy at a total dose of 50 Gy in 25 fractions.

However, the use of adjuvant or neo-adjuvant RT has largely gone understudied, since EMC has been considered for years a radiotherapy-resistant tumor, like conventional chondrosarcoma. Available data are limited and do not allow us to draw definitive conclusions. Therefore, the general recommendations about the use of adjuvant or neo-adjuvant RT in soft tissue sarcoma also apply to EMC: wide surgical margin resection and radiotherapy are standard for all sarcomas greater than 5 cm, deep and intermediate/high-grade lesions, as reported in the European Society of Medical Oncology (ESMO) and National Comprehensive Cancer Network (NCCN) guidelines [49,51]. That said, a growing interest about the use of radiotherapy in EMC has been seen in recent years, in light of a potential local control benefit. A retrospective study of 87 patients with EMC reported a better local control in patients with localized disease treated with surgery associated with radiation therapy [52]. In this series, 30% of patients were treated with wide excision and neo-adjuvant or intra-operative or adjuvant radiotherapy while 70% received surgery alone, with a trend of a better local recurrence rate in patients treated with surgery combined with radiotherapy. This observation was confirmed by another retrospective series including 41 EMC patients from a large tertiary US institution that also showed a better local control with surgery and radiation compared to surgery alone (10-year local control rate of 100% vs. 63%) [53]. In 2016, the first population-based analysis of the Surveillance, Epidemiology, and End Results (SEER) database investigated the survival outcome in 156 patients with localized EMC diagnosed between 2004 and 2012 receiving radiation therapy [54]. Ninety-four percent of patients received surgery and 32% received external beam radiotherapy (29% of them received radiation combined with surgical resection). Again, at a median follow-up of 33 months, a cancer-specific survival at 3 and 5 years in favor of radiotherapy was observed (97% vs. 85% and 94% vs. 85%, respectively), suggesting that in localized disease, surgical resection associated with radiation therapy could be considered, particularly for large lesions, when tumor shrinkage is needed for surgical down-staging or in case of loco-regional lymph node involvement (Figure 3) [49,51].

Finally, another retrospective analysis from three Italian sarcoma referral institutions that, for the first time, included only localized and molecularly confirmed cases, reported on 67 patients and found a 5-year disease-free survival of only 51% after complete resection but with a prolonged overall survival (median overall survival not reached at a median follow-up of 55 months) [14]. The 5- and 10-year local recurrence-free survival was 86% and 71%, respectively, with a 5- and 10-year distant metastases-free survival of 64% and 35%. In this series, 23 (18%) patients received surgery combined with radiation therapy, either in neo-adjuvant or in an adjuvant setting, with a better local recurrence-free survival than patients that did not receive any radiation therapy. A slightly better distant metastases-free survival was seen in patients carrying EWS-NR4A3 compared to TAF15-NR4A3 translocation.

Indeed, the metastatic risk on the long run is also high. However, distant metastases may be indolent and patients may survive for many years even with no treatment. A multi-institutional series of 42 cases of EMC from Japan showed a disease-free survival of 45% at 5 years and 36% at 10 years. Thirty patients in this series were treated with wide local excision for localized disease, showing a recurrence-free survival of 84% and 55% and a disease-free survival of 61% and 31% at 5 and 10 years, respectively [50].

Two large retrospective series of EMC, mostly treated for primary localized disease [5,52], showed 5-, 10-, and 15-year survival rates of 82–90%, 65–70%, and 58–60%, respectively, with older age, tumor size, proximal location, and high grade (increased cellularity and atypia) being adverse prognostic factors [5,55].

Despite the high rate of distant recurrence after radical surgery, no data are available to support the use of (neo) adjuvant chemotherapy in EMC [49,50,52,56].

## 4. State of the Art: Advanced Disease

The metastatic rate observed after radical surgery in EMC ranges from 25 to 50% [3,4,16,17,50,52,55]. However, prolonged survival is common even in the presence of metastatic disease. Unfortunately, data on post-distant relapse survival in EMC are scanty. Recently, a retrospective study of a small series of EMC patients detected a 5-year post-relapse survival (PRS) of 100% and 92% in cases who achieved and who did not achieve a complete surgical remission of the metastatic lesions, respectively. More than 80% of the completely resected cases suffered of a new distant relapse and the time to further recurrence shortened over time, suggesting that EMC, although indolent, tends to become more aggressive throughout its natural history [14]. Unfortunately, metastatic patients who cannot be resected eventually die of disease.

The role of surgery in the advanced phase has never been specifically studied in this sarcoma subtype. As such, it follows the algorithm usually in place for all other STS. However, it is worth noting that while EMC is often a slow-growing disease, and therefore surgery/ablation of isolated metastases is something to always consider, metastatic EMC may remain stable for many months and sometimes years. It is therefore important to also factor the pace of growth into the decision for a local therapy. Besides surgical resection, definitive radiotherapy in the various available modalities can be a valid alternative.

Metastatic patients with non-resectable disease and with evidence of tumor progression need a systemic therapy. In the past, EMC was regarded as equivalent to conventional chondrosarcoma and therefore considered refractory to cytotoxic agents commonly used for the treatment of sarcoma. The limited data available from older retrospective series showed almost no radiologic responses in patients with EMC treated with anthracycline-based, dacarbazine-based, and ifosfamide-based regimens [14,52,55,57,58]. Only two of six metastatic EMC patients responded to a multi-agent chemotherapy in the series published by McGrory et al. Of note, in all these series, the pathologic diagnosis of EMC was not molecularly supported by the detection of NR4A3 translocation events. More recently, a small retrospective series including 10 molecularly confirmed EMC, collected within the Italian Rare Cancer Network, showed a higher sensitivity to anthracycline-based regimens compared to what was previously reported, with four partial responses and a median progression-free survival of 8 months [59].

Besides doxorubicin, a phase III randomized trial of trabectedin vs. best supportive care in translocation-related sarcoma showed a prolonged disease stabilization (over 1 year) in a patient with metastatic EMC [43], suggesting that trabectedin may also be considered as an option. To the best of our knowledge, no data are available on the efficacy of gemcitabine. Besides cytotoxics, antiangiogenic agents represent a promising alternative systemic treatment, with an antitumor effect that is superior to that seen in the majority of other STSs [39,40,42,60,61,62].

The first data were on sunitinib, with a retrospective study describing six Response Evaluation Criteria in Solid Tumor (RECIST) partial responses of 10 *NR4A3*-positive EMCs; interestingly all patients who responded showed the *EWSR1-NR4A3* fusion gene whilst no activity was observed in the two *TAF15-NR4A3*-positive patients included in the series [39,42] (Figure 1C–F).

This study was followed by a prospective European phase II single-arm trial on pazopanib in *NR4A3*-positive advanced EMC (NCT02066285) [40]. Twenty-six EMC patients were enrolled; at a median follow-up of 27 months, four of 22 evaluable patients had a RECIST partial response (all of them with *EWSR1-NR4A3* fusion), and 55% of the cases showed a certain degree of tumor shrinkage, even though <30% was required by RECIST. The disease control was prolonged, with a median progression-free survival of 19 months, while the median overall survival was not reached.

Although the precise mechanism behind the selective activity of antiangiogenics in EMC is still undefined, the transcriptional profiling of a subset of responder and non-responder EMCs highlighted up-regulation of canonical pazopanib targets FMS-lyke tyrosine kinase 1 (FLT1/vascular endothelial growth factor receptor 1-VEGFR1), kinase insert domain receptor (KDR/vascular endothelial growth factor receptor 2-VEGFR2), FMS-lyke tyrosine kinase receptor 4 (FLT4/vascular endothelial growth factor receptor 3-VEGFR3), and cognate ligands (vascular endothelial growth factor A-VEGFA and vascular endothelial growth factor C-VEGFC), as well as components of the Notch Homolog Protein (NOTCH) pathway in the pazopanib-sensitive cohort. Intriguingly, as observed with sunitinib, all three *TAF15-NR4A3*-positive tumors included in this pazopanib trial failed to respond to pazopanib, which suggests that the more malignant phenotype of the TAF15-translocated EMC may impair antiangiogenic sensitivity [35]. Eventually, a retrospective study of apatinib, a new selective inhibitor of VEGFR2 under investigation in different cancers, among which is STS, identified one partial response and two stable diseases of three EMC patients, with an interesting median duration of response of 21.2 months [61]. Of note, pazopanib is the only antiangiogenic agent formally approved for EMC treatment, from second line and restricted only to adult patients.

Insulin-like growth factor receptor 1 (IGFR1) inhibitors also showed a certain degree of clinical activity in EMC [63]. Unfortunately, the clinical development of this class of agents was discontinued based on disappointing results in other more frequent cancers [44,64,65,66,67].

## 5. Current Research and Future Perspectives

EMC displays an interesting immune infiltration pattern [40], however, only very preliminary data are currently available on the activity of programmed cell-death protein 1/programmed death-ligand 1 (PD1/PDL1) inhibitors in EMC [68]. The Spanish and the Italian Sarcoma Group recently conducted a phase I–II single-arm trial (NCT03277924) with the combination of sunitinib and nivolumab in several histotypes of soft tissue and bone sarcomas observing, among others, one RECIST and two Choi partial responses out of four patients with EMC enrolled in the trial [62]. However, the study design does not allow for understanding what has been contributed in terms of antitumor effect by the addition of nivolumab to sunitinib.

Among new epigenetic therapies, tazemetostat, an enhancer of zeste homologue 2 (EZH2) inhibitor, recently approved by the US Food and Drug Administration (FDA) for the treatment of advanced epithelioid sarcoma, has also been tried in integrase interactor 1 (INI-1) negative EMC cases [69] within a phase I–II basket trial of INI-1-negative solid tumors (NCT02601937, NCT02601950), with pending results.

Overall, EMC patients who suffer distant relapse have an unfavorable prognosis and medical options available in these cases are limited, although the survival in the advanced phase is often longer than expected in other STS. Although in vitro investigations and tumor molecular profiling studies are suggesting novel potential therapeutic targets [10,35,45,46] (Table 1), there is a strong urgency to identify prognostic factors and therapeutic strategies to improve the chance of a cure at disease onset and in case of relapse. To this end, networking is essential, as well as fostering discussions with the regulators on how to improve research and drug development in ultra-rare tumors.

## 6. Conclusions

EMC rarity makes clinical research very challenging for this disease. However, EMC is an example of how prospective studies can also be conducted in ultra-rare settings within a network of collaboration, as shown by the above-mentioned phase II trial of pazopanib, which enrolled 26 patients in three years. In addition, EMC shows that the knowledge of the biological behavior and natural history of a rare condition can be investigated by retrospective studies/case series, provided that they include patients whose pathologic diagnosis is confirmed by expert sarcoma pathologist review and molecular assessment. Of course, the identification of a disease-specific molecular alteration in EMC to be used as a tracer has contributed a lot in this respect. The results collected to date are also important to provide a benchmark for external comparison with prospective single-arm studies on new treatment approaches, considering how randomized trials are difficult to conceive in ultra-rare tumors.

## Figures and Tables

**Table 1:** Main ongoing or recently completed trials on sarcoma, also open to extraskeletal myxoid chondrosarcoma patients (updated July 2020).

Study Title	Phase	Status	Conditions	Age at Study Entry	ClinicalTrials.Gov Identifiers
Phase I–II trial of sunitinib plus nivolumab after standard treatment in advanced soft tissue and bone sarcomas	I–II	Recruiting	Advanced STS Advanced BS	12–80 yrs	NCT03277924
A phase 1 study of the EZH2 inhibitor tazemetostat in paediatric subjects with relapsed or refractory INI1-negative tumors or synovial sarcoma	I	Recruiting	Advanced SS Advanced INI1-negative tumors	6 mos–18 yrs	NCT02601937
A phase II, multicenter study of the EZH2 inhibitor tazemetostat in adult subjects with INI1-negative tumor or relapsed/refractory synovial sarcoma	II	Recruiting	Advanced SS Advanced INI1-negative tumors	≥18 yrs	NCT02601950
A phase II open-label trial of pazopanib administered as a single agent in patients with unresectable or metastatic solitary fibrous tumour (SFT) or extraskeletal myxoid chondrosarcoma (EMC)	II	Completed	Advanced SFT Advanced EMC	≥18 yrs	NCT02066285
A phase II trial of perifosine in patients with chemo-insensitive sarcomas: Sarcoma Alliance for Research through Collaboration (SARC) multi-center trial	II	Completed	Advanced CS Advanced ASPS Advanced EMC	≥13 yrs	NCT00401388
A Phase II trial of R1507, a recombinant human monoclonal antibody to the insulin-like growth factor-1 receptor for the treatment of participants with recurrent or refractory Ewing’s sarcoma, osteosarcoma, synovial sarcoma, rhabdomyosarcoma and other sarcomas	II	Completed	Advanced STS Advanced BS	≥2 yrs (Ewing’s sarcoma cohort 2–21 yrs)	NCT00642941
Pazopanib neoadjuvant trial in non-rhabdomyosarcoma soft tissue sarcomas (PAZNTIS): A phase II/III randomized trial of preoperative chemoradiation or preoperative radiation plus or minus pazopanib	II–III	Active, not recruiting	Localized STS	≥2 yrs	NCT02180867
A phase 1 study of doxorubicin and A12 (cixutumumab) in advanced soft tissue sarcoma	I	Completed	Advanced STS	≥16 yrs	NCT00720174
A phase 1B/II study of GDC-0449 (NSC 747691-vismodegib) in combination with RO4929097, a gamma-secretase Inhibitor (GSI) in advanced/metastatic sarcomas	I–II	Completed	Advanced STS	≥18 yrs	NCT01154452
A randomized, double-blind phase II, study of Gemcitabine alone or in combination with pazopanib for refractory soft tissue sarcoma	II	Active, not recruiting	Advanced STS	≥18 yrs	NCT01532687

EMC: extraskeletal myxoid chondrosarcoma; CS: chondrosarcoma; ASPS: alveolar soft part sarcoma; mos: months; yrs: years.
